# 79. Children with COVID-19 Demonstrate Distinct Serum Cytokines Profiles According to Clinical Presentations

**DOI:** 10.1093/ofid/ofab466.079

**Published:** 2021-12-04

**Authors:** Zhaohui Xu, Rebecca M Glowinski, Shira H Cohen, Cameron Mertz, Sara Mertz, Fang Ye, Pablo J Sanchez, Asuncion Mejias, Octavio Ramilo

**Affiliations:** 1 Nationwide Children's Hospital, Columbus, OH; 2 columbus, Ohio; 3 Nationwide Children's Hospital - The Ohio State University, Columbus, Ohio

## Abstract

**Background:**

Almost 4 million children have tested positive for Coronavirus Disease 2019 (COVID-19) as of June 3 2021, representing 14% of all cases in USA. Children present with diverse clinical findings including the multisystem inflammatory syndrome in children (MIS-C). In this study, we measured serum cytokine concentrations in children with COVID-19 to identify differences in immune profiles according to clinical presentations.

**Methods:**

A total of 133 children 0-21 years of age with COVID-19 were enrolled at Nationwide Children’s Hospital, in Columbus, Ohio. Nasopharyngeal swab RT-PCR testing was used for SARS-CoV-2 detection and quantification. Clinical and laboratory information were obtained, and blood samples were collected for measurement of cytokines with a 92-plex inflammation assay (Olink). Normalized cytokine expression levels in patients were compared with serum samples from 66 pre-pandemic age-matched healthy controls.

**Results:**

COVID-19 children included: 1) those identified by universal screening (n=47); 2) moderate disease (ward; n=48); 3) severe disease (PICU; n=20); 4) MIS-C (n=18). Children identified by universal screening were hospitalized for trauma, appendicitis or new onset diabetes among others. Children with symptomatic COVID-19 had significantly higher SARS-CoV-2 viral loads than children with MIS-C or those identified via universal screening. Concentrations of interferon (IFN) related cytokines (IFNg, CXCL9, CXCL10, CXCL11), interleukins (IL6, IL8, IL10, IL17A, IL18, IL24) and other inflammatory cytokines (TGF, TNF, VEGF, MCP, CD40) were significantly increased in children with acute COVID-19 and MIS-C compared with children identified by universal screening and healthy controls. These cytokines were positively correlated with C-reactive protein, D-dimer and disease severity in COVID-19, but negatively correlated with viral loads (Fig 1). MIS-C showed stronger inflammatory response than acute COVID-19 (Fig 2).

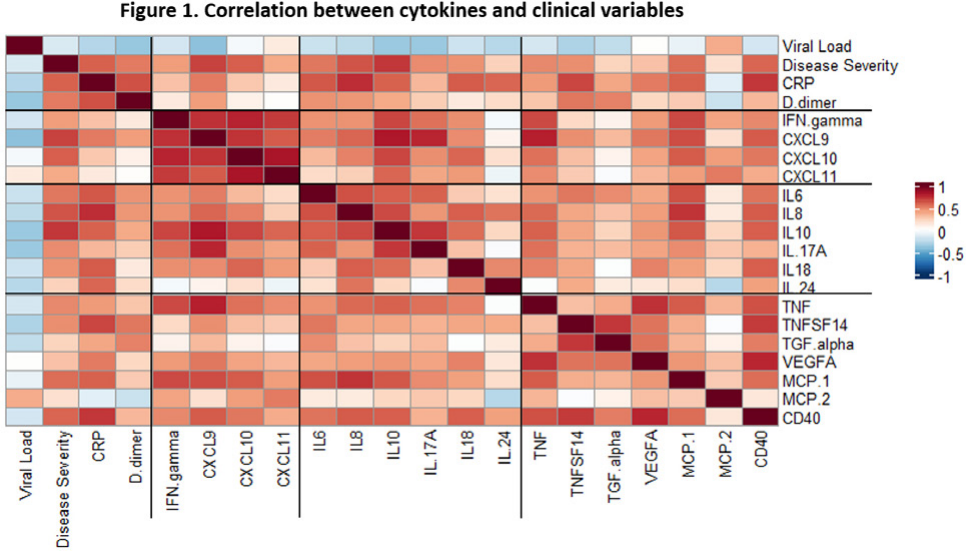

Correlation of Age-adjusted cytokine expression values with viral load, disease severity, CRP and D-dimer. Pearson correlation coefficient is shown for each pair. Red: positive correlation; blue: negative correlation

Cytokines that differentiate MIS-C from acute COVID-19

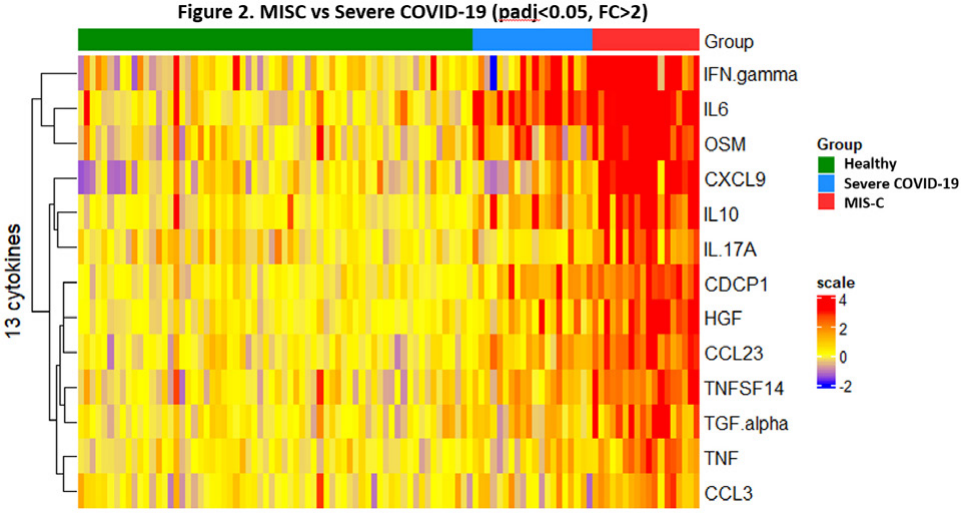

Heatmap shows the differential expressed cytokines between MIS-C and acute severe COVID-19 (padj<0.05, FC>2). The age-adjusted expression values are normalized the median of healthy controls. Red: up-regulation, blue: down-regulation.

**Conclusion:**

We identified three cytokine clusters in children with COVID-19 according to clinical presentations. Correlations of serum cytokines with clinical/laboratory parameters could be used to identify potential biomarkers associated with disease severity in COVID-19

**Disclosures:**

**Asuncion Mejias, MD, PhD, MsCS**, **Janssen** (Grant/Research Support, Advisor or Review Panel member)**Merck** (Grant/Research Support, Advisor or Review Panel member)**Roche** (Advisor or Review Panel member)**Sanofi** (Advisor or Review Panel member) **Octavio Ramilo, MD**, **Adagio** (Consultant)**Bill & Melinda Gates Foundation** (Grant/Research Support)**Janssen** (Grant/Research Support)**Lilly** (Consultant)**Merck** (Consultant, Grant/Research Support)**NIH** (Grant/Research Support)**Pfizer** (Consultant)**SANOFI** (Board Member)

